# SNORA72 Activates the Notch1/c-Myc Pathway to Promote Stemness Transformation of Ovarian Cancer Cells

**DOI:** 10.3389/fcell.2020.583087

**Published:** 2020-11-03

**Authors:** Liwen Zhang, Rong Ma, Mengcong Gao, Yanyun Zhao, Xuemei Lv, Wenjing Zhu, Li Han, Panpan Su, Yue Fan, Yuanyuan Yan, Lin Zhao, Heyao Ma, Minjie Wei, Miao He

**Affiliations:** ^1^Department of Pharmacology, School of Pharmacy, China Medical University, Shenyang, China; ^2^Liaoning Key Laboratory of Molecular Targeted Anti-Tumor Drug Development and Evaluation, China Medical University, Shenyang, China; ^3^Liaoning Cancer Immune Peptide Drug Engineering Technology Research Center, China Medical University, Shenyang, China; ^4^Key Laboratory of Precision Diagnosis and Treatment of Gastrointestinal Tumors, Ministry of Education, China Medical University, Shenyang, China

**Keywords:** SNORA72, ovarian cancer stem cells (OCSCs), Notch1, c-Myc, stemness

## Abstract

Cancer stem cells (CSCs) are responsible for the migration and recurrence of cancer progression. Small nucleolar RNAs (snoRNAs) play important roles in tumor development. However, how snoRNAs contribute to the regulation of the stemness of ovarian CSCs (OCSCs) remains unclear. In the present study, we found that SNORA72 was significantly upregulated in OVCAR-3 spheroids (OS) and CAOV-3 spheroids (CS) with the OCSC characteristics attained by serum-free culture in a suspension of OVCAR-3 (OV) and CAOV-3 (CA) cells. The overexpression of SNORA72 increased self-renewal abilities and migration abilities in OV and CA cells and upregulated the expressions of the stemness markers Nanog, Oct4, and CD133. In addition, the ectopic expression of SNORA72 can elevate the messenger RNA (mRNA) and protein expression levels of Notch1 and c-Myc in parental cells. The opposite results were observed in SNORA72-silenced OCSCs. Moreover, we found that Notch1 knockdown inversed the migration abilities and self-renewal abilities raised by overexpressing SNORA72. In summary, stemness transformation of ovarian cancer cells can be activated by SNORA72 through the Notch1/c-Myc pathway. This study introduces a novel therapeutic strategy for improving the treatment efficiency of ovarian cancer.

## Introduction

Ovarian cancer has the highest mortality rate among all gynecological cancers (Siegel et al., 2020). More than 80% of patients with ovarian cancer present with advanced-stage disease when diagnosed (Narod, 2016; Li et al., 2018). It has been reported that approximately 15–25% of patients are resistant to initial platinum-based chemotherapy; even patients with an initial complete response will relapse and develop resistance (Agarwal and Kaye, 2003; Bapat et al., 2005; Zhang et al., 2014; Ottevanger, 2017). Targeting the roots of metastasis and resistance of ovarian cancer cells is the key to treating ovarian cancer. Cancer stem cells (CSCs) are rare cells in cancers with indefinite potential for self-renewal that drive tumorigenesis and recreate heterogeneity. Growing evidence shows that CSCs not only influence cancer initiation but also drive tumor progression, therapy resistance, and metastatic growth (Reya et al., 2001; Chen et al., 2017; Lytle et al., 2018). Therefore, exploring the targets and mechanisms of maintaining the stemness of ovarian CSCs (OCSCs) will provide better insights into the treatment of ovarian cancer.

Small nucleolar RNAs (snoRNAs) are one of the best-characterized classes of non-coding RNAs (ncRNAs) in the nucleus and are 60–300 nucleotides in length. They are classified as C/D box snoRNAs (SNORDs), serving as guides for 2′-*O*-ribose methylation of ribosomal RNAs (rRNAs) or small nuclear RNAs (snRNAs), and H/ACA box snoRNAs (SNORAs), guiding the isomerization of uridine residues into pseudouridine (Jorjani et al., 2016). Accumulating evidence suggests that the dysregulation of snoRNAs can function in controlling cell fate and regulating tumorigenesis and development (Gong et al., 2017; Romano et al., 2017). Cui et al. found increased SNORA23 expression in human pancreatic ductal adenocarcinoma (PDAC) tissues, and SNORA23 promoted the survival and invasion of PDAC cells (Cui et al., 2017). SNORA21 was systematically identified as a key oncogenic snoRNA in colorectal cancer (CRC), which might serve as a prognostic biomarker (Yoshida et al., 2017). In recent years, the impact of snoRNAs on the characteristics of CSCs has gained more and more attention. SNORA42 has been reported to contribute to the self-renewal and proliferation abilities of lung tumor-initiating cells (Mannoor et al., 2014). However, abnormal snoRNAs in OCSCs and their functions have rarely been reported.

In this study, we found that SNORA72 was highly expressed in ovarian sphere cells with OCSC-like characteristics. The ectopic expression of SNORA72 increased the self-renewal and migration abilities of ovarian cancer cells. Conversely, the knockdown of SNORA72 inhibited the self-renewal and migration abilities of OCSCs. Furthermore, we demonstrated that SNORA72 might maintain the stemness of OCSCs by activating the Notch1/c-Myc pathway.

## Materials and Methods

### Induction of Ovarian Cancer Spheroid Cells

A method for inducing cancer cells to CSCs was reported previously (Zhong et al., 2010; Wang et al., 2012; He et al., 2019). In brief, OVCAR-3 (OV) (RRID:CVCL_0465) and CAOV3 (CA) (RRID:CVCL_0201) cells (Cell Bank of the Chinese Academy of Sciences, Shanghai, China) were cultured in RPMI-1640 medium (Hyclone, United States) with 10% fetal bovine serum (FBS) (Tian Jin Hao Yang Biological Manufacture CL., LTD, China). Tumor spheres were derived at a density of 1 × 10^5^ cell/ml from monolayers of OV and CA into a serum-free Dulbecco’s modified Eagle’s medium (DMEM)/F12 medium (Hyclone) with 10 ng/ml basic fibroblast growth factor (bFGF; Peprotech Corporation, United States), 20 ng/ml epidermal growth factor (EGF; Peprotech Corporation), and 2% B27 (Invitrogen Corporation, United States). After several generations, the cells grew into nonadherent spherical clusters termed OVCAR-3 spheres (OS) and CAOV3 spheres (CS). The cell lines used in this study did not suffer from mycoplasma infection. All cell lines were authenticated using STR profiling.

### Limiting Gradient Dilution

Single cell suspensions of OV, OS, CA, and CS cells were seeded into 96-well plates at a density of 200, 100, 50, or 25 cells/well, and each gradient has 20 wells. The number of wells with colonies in each cell gradient (Wells with Colonies) and the total number of monoclonal cell spheres in each cell gradient (Total colonies) were counted, and the percentage of the total number of monoclonal cell spheres to the total cells of each cell gradient (Total colonies/Total cells × 100%) was calculated after 9 days.

### Flow Cytometry

CD133 expression in OV and OS cells was measured by flow cytometry. The cells were collected and washed several times with phosphate-buffered saline (PBS). The cells were incubated with CD133 antibody (1:20, BD Pharmingen, United States) at 4°C for 15–20 min in the dark. After that, the cells were washed with cold PBS three times. Finally, we tested single cell suspensions on a flow cytometer (ACEA Biosciences Inc., China).

### Quantitative Reverse Transcription PCR

Total RNA was extracted using the TRIzol reagent, and snoRNAs were transcribed into complementary DNA (cDNA) in a 10-μl reaction volume, which contains 2 μl 5× M-MLV buffer, 0.3 μl M-MLV (200 U/μl), 0.25 μl RNase inhibitor (40 U/μl), 1 μl dNTP (1 U/μl), 1 μl RT-primer (5 μM), and 5.45 μl RNA (200 ng/μl) at 42°C for 60 min, 70°C for 10 min, and then kept at 4°C. The messenger RNAs (mRNAs) of other genes reacted in 10 μl volume with 2 μl 5× RT, 0.5 μl enzyme mix, 0.5 μl primer mix, and 7 μl RNA at 37°C for 15 min, 9°C for 5 min, and then kept at 4°C. Specific SNORA72 primer sequences were designed by RiboBio (Guangzhou, China) and small nuclear RNA U6 was used as the internal control. The mRNA expression levels were normalized to that of β-actin. The primer sequences were: β-actin forward 5′-TCCTCCCTGGAGAAGAGCTA-3′, reverse 5′-TCCTGCTTGCTGATCCACAT-3′; Oct4 forward 5′-CTTGCTGCAGAAGTGGGTGGAGGAA-3′, reverse 5′-CTG CAGTGTGGGTTTCGGGCA-3′; Nanog forward 5′AATACCT CAGCCTCCAGCAGATG-3′, reverse 5′-TGCGTCACACCATT GCTATTCTTC-3′; c-Myc forward 5′-CGACGAGACCTTC ATCAAAAAC-3′, reverse 5′-CTTCTCTGAGACGAGCTTGG-3′; and CD133 forward 5′-GTGGCGTGTGCGGCTATGAC-3′, reverse 5′-CCAACTCCAACCATGAGGAAGACG-3′. The 2^–ΔΔCt^ method was used to calculate the relative fold.

### Reverse Transcription Polymerase Chain Reaction

The reverse transcription process was consistent with the quantitative reverse transcription PCR (qRT-PCR) process. After the reverse transcription product was diluted 10 times, it was diluted with 5 μl Taq enzyme, 0.5 μl upstream primer, 0.5 μl downstream primer, and 4 μl cDNA to form a 10-μl reaction volume in the following procedure: 98°C for 10 s; 60°C for 30 s; 72°C for 60 s, 25 cycles for snoRNA reaction, and 30 cycles for mRNA reaction.

### Transfection

SNORA72-cDNA plasmids (Shanghai Genechem Co., LTD, China) were used to transfect the OV and CA cells in a six-well plate to overexpress SNORA72. The shRNA-SNORA72 plasmids (Shanghai Genechem Co., LTD) were transfected into the OS and CS cells seeded in six-well plates to inhibit SNORA72 expression. shRNA-Notch1 plasmids (Shanghai Genechem Co., LTD) were used to transfect OV and CA cells to inhibit the expression of Notch1. Lipofectamine 3000 (Invitrogen) was used for transfection according to the manufacturer’s instructions.

### Plate Clone Formation Assay

The cells transfected with the indicated plasmids for 48 h were digested using 0.25% trypsin and pipetted into single cells. The resuspended single cells were seeded in a six-well plate (1,000 cells per well) and placed in an incubator at 37°C, 5% CO_2_ atmosphere, and saturated humidity for 2–3 weeks. Afterward, the cells were washed with PBS two to three times, and the cells were fixed with 1 ml 4% paraformaldehyde in each well. The cells were stained with 0.5% crystal violet (Sigma-Aldrich 46364) for 30 min and then washed until the background color was clean. Pictures were taken and the number of colonies were counted using a Nikon eclipse TE2000-U microscope.

### Sphere Formation Assays

After transfection with the indicated plasmids for 48 h, OS and CS cells were cultured in ultra-low adherent six-well plates with a density of 2,000 cells/well in DMEM-F12 medium (HyClone) containing 10 ng/ml bFGF (Peprotech Corporation), 20 ng/ml EGF (Peprotech Corporation), and 2% B27 (Invitrogen Corporation). After culturing for 14 days, spheres with a diameter >150 μm were counted.

### Bioinformatics Analysis

Kaplan–Meier survival curves of progression-free survival (PFS) for SNORA72 were produced using an online tool called Kaplan–Meier Plotter^1^, and 1,435 patients were included for analysis. We performed gene correlation analysis using the cBioPortal^2^ and the online tool R2: Genomics Analysis and Visualization Platform^3^.

### Transwell Assay

The upper chambers of Transwell plates precoated with Matrigel (Corning, United States) were plated with 1 × 10^4^ OV or CA cells in 500 μl serum-free RPMI-1640 medium or 1 × 10^4^ OS or CS cells in 500 μl serum-free DMEM/F12 medium. The lower chambers were filled with 500 μl medium containing 10% FBS. Then, the cells were allowed to migrate toward the lower chambers for 48 h in an atmosphere of 5% CO_2_ at 37°C. The chambers were then fixed with methanol and stained with 0.1% crystal violet. The number of cells migrating across the membrane were counted under a light microscope and analyzed using ImageJ software.

### Wound-Healing Assays

Cells were cultured in six-well plates until 70% confluency. OV and CA cells were transfected with SNORA72-NC, SNORA72-OE, SNORA72-OE+sh-NC, and SNORA72-OE+sh-Notch1. Linear “scratches” were created on the monolayer cells in straight lines with sterile tips. The cells were washed three times with PBS and serum-free medium was added. The cells were photographed after 0, 24, and 48 h of incubation under a microscope (Nikon Eclipse TE2000-U, Japan). Wound closure was quantified using ImageJ software.

### Western Blot

The cells were inoculated into RIPA lysis buffer containing 1% proteinase inhibitor cocktail solution and 1% phosphatase inhibitor cocktail solution. Extracted protein samples were loaded on SDS-polyacrylamide separating gel and transferred onto PVDF membranes (Millipore). The primary antibodies used were c-Myc (1:1,000, Cell Signaling Technology, United States), Notch1 (1:1,000, Cell Signaling Technology), and β-actin (1:1,000, Absin Bioscience Inc., China). Protein expression was quantitatively analyzed using Scion Image Software (Scion Corp., Frederick, MA, United States).

### Statistical Analysis

Statistical analyses were performed using the GraphPad Prism 7.0 software. Differences between two groups were statistically analyzed using Student’s *t* test, which was considered statistically significant when the *P* value was <0.05.

## Results

### SNORA72 Is Highly Expressed in OCSCs

**FIGURE 1:**
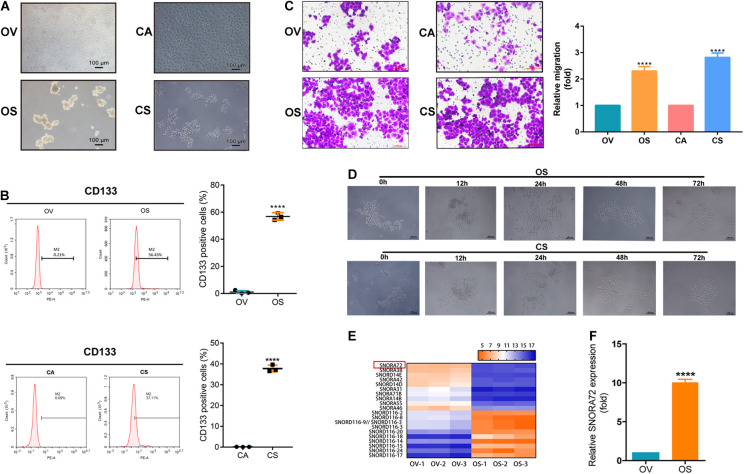
SNORA72 is overexpressed in ovarian cancer stems cells (OCSCs). **(A)** Morphology of OVCAR-3 (OV), OVCAR-3 spheroid (OS), CAOV-3 (CA), and CAOV-3 spheroid (CS) cells shown under a microscope (×10). **(B)** Expression of CD133 detected by flow cytometry in OV vs OS and in CA vs CS cells. **(C)** Migration abilities of OV, OS, CA, and CS cells by Transwell assay. **(D)** Differentiation morphology of OS and CS cells at 0, 12, 24, 48, and 72 h. **(E)** Hierarchical clustering analysis of small nucleolar RNA (snoRNA) expression from non-coding RNA-ChIP data in OV and OS cells. *Red*, higher expression levels; *green*, lower expression levels. **(F)** Relative SNORA72 expression to U6, as an endogenous control, analyzed by qRT-PCR in OV and OS cells. The SNORA72 expression in OV cells was set as 1. Data are shown as the mean ± SD from three independent experiments. **P < 0.01, ****P < 0.0001.

We induced and successfully enriched OCSCs according to section “Induction of Ovarian Cancer Spheroids Cells” and our previous reports (He et al., 2019; Zhu et al., 2019). As shown in Figure 1A, the OS and CS had a dense spherical morphology. The expression of CD133, an OCSC marker, was found to be higher in OS and CS than in its parental OV and CA cells by flow cytometry (Figure 1B). In addition, we also compared the monoclonal cell sphere formation ability of OV vs OS as well as CA vs CS cells through limiting gradient dilution analysis. As shown in Table 1, under each cell dilution gradient, the proportion of monoclonal spheres in OS and CS cells is higher than in that OV and CA cells, indicating that spheroid cells have a strong ability to form monoclonal spheres. The Transwell assay showed that OS and CS had significantly higher number of cells migrating across the membrane than did OV and CA (Figure 1C). Moreover, in order to identify the differentiation potential of OS and CS cells, we transferred serum-free non-adherent cultured OS and CS cells into 1640 medium with 10% FBS. OS and CS cells began to grow adherently at 12 h and had completely lost the characteristics of spheroids at 72 h (Figure 1D). Therefore, these results suggested that OS and CS cells had stem cell properties.

**TABLE 1:** Limited gradient dilution analysis experiment for OV, OS, CA and CS.

	OV				OS				CA				CS			
Dilution ratio	1/1	1/2	1/4	1/8	1/1	1/2	1/4	1/8	1/1	1/2	1/4	1/8	1/1	1/2	1/4	1/8
Cells/well	200	100	50	25	200	100	50	25	200	100	50	25	200	100	50	25
Wells with colonies	12	8	7	3	20	20	18	16	14	9	7	4	20	20	18	15
Total colonies	163	49	21	9	1,215	559	235	107	176	54	25	11	1,179	535	233	113
Total cells	4,000	2,000	1,000	500	4,000	2,000	1,000	500	4,000	2,000	1,000	500	4,000	2,000	1,000	500
Colonies/total cells (%)	4.07	2.45	2.1	1.8	30.37	27.95	23.5	21.4	4.4	2.7	2.5	2.2	29.48	26.75	23.3	22.6

*OV, OVCAR-3 cells; OS, OVCAR-3 spheroids cells; CA, CAOV-3 cells; CS, CAOV-3 spheroids cells.*

In order to explore the factors that affect the maintenance of the stemness of OCSCs, we performed non-coding RNA chromatin immunoprecipitation (ChIP) analysis of OV and OS cells. Interestingly, we found that a large number of snoRNAs were abnormally expressed between OV and OS. We speculated that snoRNAs might play major roles in the stemness phenotype transformation of ovarian cancer cells. Further analysis revealed that SNORA72 was highly expressed in OS cells compared to OV cells (Figure 1E). Consistent with this, we observed that SNORA72 expression was 10.06 ± 0.392-fold in OS cells relative to OV cells by qRT-PCR analysis (Figure 1F). Thus, the data indicate that it is necessary to explore the effects of SNORA72 on stemness maintenance in ovarian cancer cells.

### SNORA72 Maintains Self-Renewal of OCSCs

**FIGURE 2:**
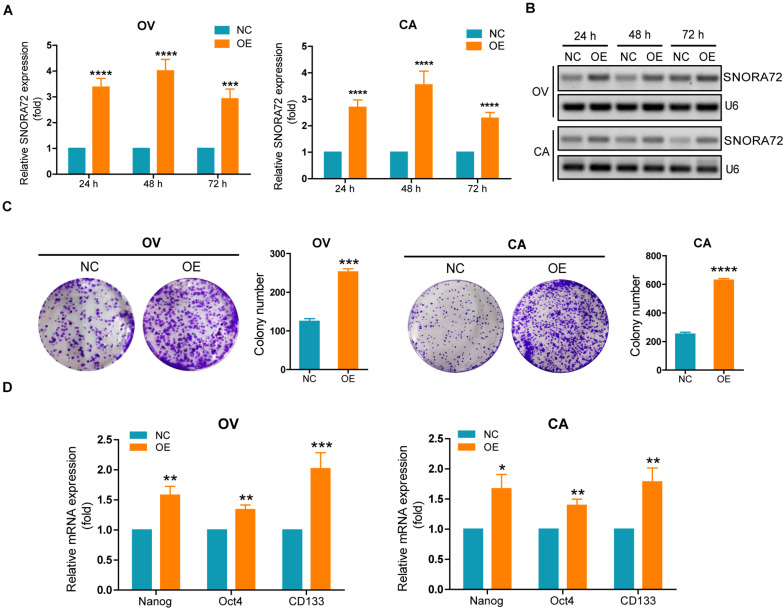
SNORA72 overexpression increases the self-renewal of OVCAR-3 (OV) and CAOV-3 (CA) cells. Expression of SNORA72 determined in OV and CA ovarian cells at 24, 48, and 72 h after transfection of the SNORA72 overexpression (OE) or negative control (NC) plasmids by **(A)** quantitative reverse transcription PCR (qRT-PCR) and **(B)** RT-PCR analysis. **(C)** Changes of the clone performing ability detected by the plate clone formation assays in OV and CA cells transfected with the OE or NC plasmids. **(D)** Relative mRNA expressions of Nanog, Oct4, and CD133 to β-actin, as an endogenous control, analyzed by qRT-PCR in OV and CA cells after transfection of the OE or NC plasmids for 48 h. The expression level in OV and CA cells after transfection of NC plasmids was set as 1. *P < 0.05, **P < 0.01, ***P < 0.001, and ****P < 0.0001.

In order to investigate the effects of SNORA72 on the stemness phenotype transformation of ovarian cancer cells, we first transfected SNORA72-cDNA plasmids into OV and CA cells to evaluate changes in their self-renewal abilities. SNORA72 expression levels in OV cells transfected with SNORA72-cDNA plasmids (OE) after 24, 48, and 72 h were upregulated 3. 38-, 4. 01-, and 2.93-fold, respectively, by qRT-PCR analysis compared to the control (NC). In CA cells, SNORA72 expression levels in the OE groups were 2. 69-, 3. 55-, and 2.29-fold, respectively, compared to those in the NC groups after 24, 48, and 72 h transfection (Figure 2A). Similarly, agarose electrophoresis after RT-PCR also showed an increased expression of SNORA72 in OE ovarian cancer cells (Figure 2B). Next, we detected changes in the clone performing ability after SNORA72 overexpression by plate clone formation assays. We found that the overexpression of SNORA72 markedly increased the number of clones and the size of OV and CA cells (Figure 2C). In addition, we observed that the mRNA expressions of the stemness biomarkers CD133, Nanog, and Oct4 (Zhu et al., 2019) were significantly increased in OV and CA cells after the overexpression of SNORA72 (Figure 2D). The expression levels of CD133 at different times are shown in Supplementary Figures S1A,B. The results show that the expression levels of CD133 can be increased by the overexpression of SNORA72.

**FIGURE 3:**
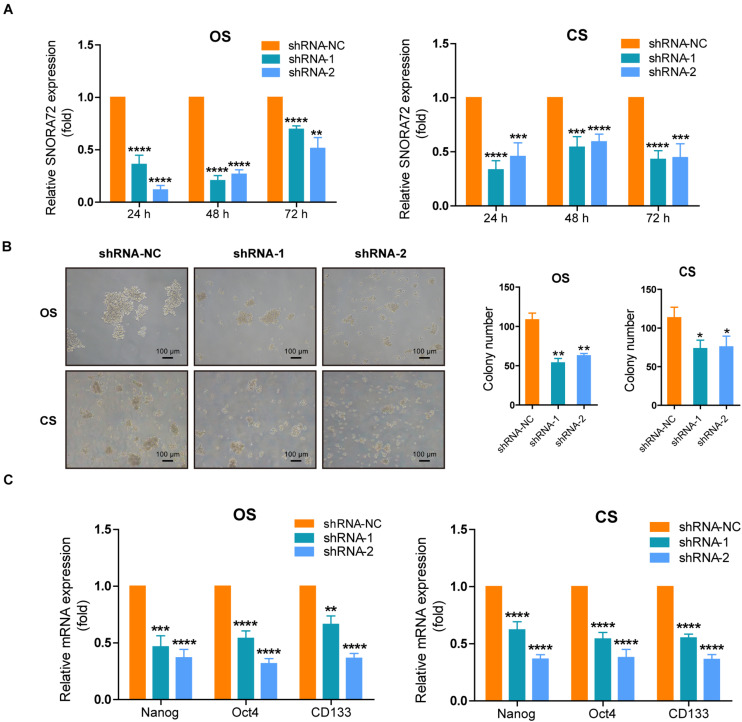
SNORA72 knockdown decreases the self-renewal of OVCAR-3 spheroid (OS) and CAOV-3 spheroid (CS) cells. **(A)** Expression of SNORA72 determined in OS and CS cells at 24, 48, and 72 h after transfection of the SNORA72 silencing (shRNA-1 and shRNA-2) or control (shRNA-NC) plasmids by quantitative reverse transcription PCR (qRT-PCR) analysis. **(B)** Self-renewal abilities were measured by colony formation assays in OS and CS cells transfected with the shRNA-1, shRNA-2, or shRNA-NC plasmids. **(C)** Relative mRNA expressions of Nanog, Oct4, and CD133 to β-actin, as an endogenous control, analyzed by qRT-PCR in OS and CS cells after transfection of the shRNA-1, shRNA-2, or shRNA-NC plasmids for 48 h. The expression level in OS and CS cells after transfection of shRNA-NC plasmids was set as 1. *P < 0.05, **P < 0.01, ***P < 0.001, and ****P < 0.0001.

We also successfully constructed SNORA72 knockdown ovarian cancer cells. Transfection of sh-SNORA72 plasmids after 24, 48, and 72 h notably reduced SNORA72 expression in both OS and CS cells (Figure 3A). Additionally, sphere formation assays showed that the knockdown of SNORA72 markedly decreased the number of spheres of OS and CS cells (Figure 3B). We also found that the expressions of CD133, Nanog, and Oct4 mRNAs were significantly downregulated in sh-SNORA72-transfected OS and CS cells than in sh-NC-transfected cells (Figure 3C). The expression levels of CD133 at different times are shown in Supplementary Figures S1C,D. The results show that the expression levels of CD133 can be decreased by the knockdown of SNORA72. All these findings suggest that SNORA72 can promote the self-renewal ability of ovarian cancer cells.

### SNORA72 Maintains the Migration Abilities of OCSCs

**FIGURE 4:**
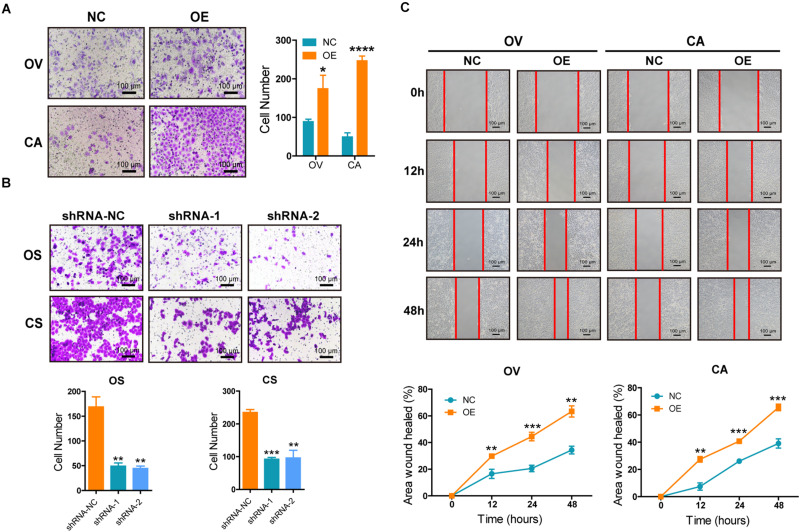
SNORA72 promotes the migration abilities of ovarian cancer cells. **(A)** Migration abilities of OVCAR-3 (OV) and CAOV-3 (CA) cells transfected with the SNORA72 transfected (OE) or control (NC) plasmids by the Transwell assay. **(B)** Migration abilities of OVCAR-3 spheroid (OS) and CAOV-3 spheroid (CS) cells transfected with the shRNA-1, shRNA-2, or shRNA-NC plasmids by the Transwell assay. **(C)** Migration abilities of OV and CA cells transfected with the SNORA72 OE or NC plasmids by the wound-healing assay. *P < 0.05, **P < 0.01, ***P < 0.001, and ****P < 0.0001.

Increased migration ability is one of the features of CSCs. We explored the effects of SNORA72 on the migration ability of OCSCs. The Transwell assays showed that the overexpression of SNORA72 significantly increased the number of cells migrating across the membrane in OV and CA cells (Figure 4A), while the number of cells migrating across the membrane in OS and CS cells after knocking down SNORA72 markedly decreased (Figure 4B). In addition, the wound-healing assays showed that the overexpression of SNORA72 significantly increased the wound-healed areas in OV and CA cells compared to the control cells. All the data suggest that SNORA72 can promote the migration abilities of ovarian cancer cells (Figure 4C).

### SNORA72 Expression Is Positively Correlated With c-Myc Expression in Ovarian Cancer Patients

**FIGURE 5:**
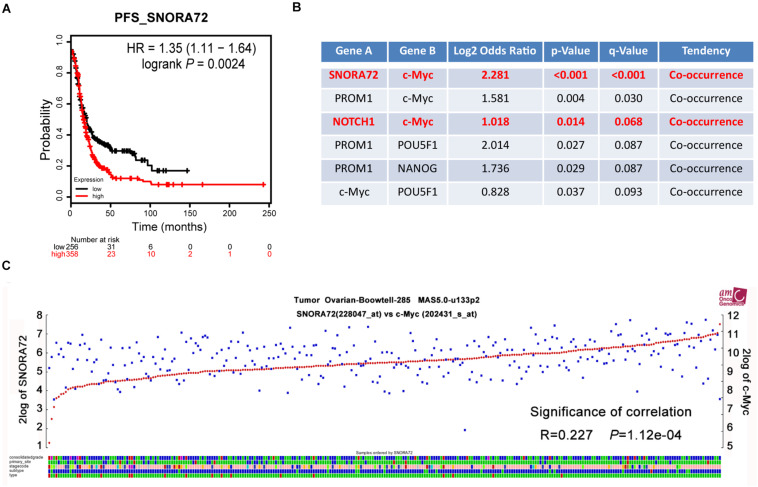
SNORA72 expression is positively associated with the c-Myc expression of ovarian cancer patients. **(A)** Online tool Kaplan–Meier Plotter used to analyze the relationship between SNORA72 expression and the progression-free survival (PFS) of ovarian cancer patients. **(B)** The relationship between SNORA72 expression and stemness markers was analyzed by the online analysis website cBioPortal. **(C)** R2: Genomics Analysis and Visualization Platform used to analyze the expression correlation between SNORA72 and c-Myc.

In order to assess the effects of SNORA72 expression on the progression of ovarian cancer patients, we used the online analysis website Kaplan–Meier Plotter to analyze the association of SNORA72 expression with PFS in ovarian cancer patients. We found that patients with a high SNORA72 expression had a shorter PFS time (HR = 1.35, *P* = 0.0024; Figure 5A). These findings suggest that SNORA72 has a higher degree of correlation with poor prognosis in ovarian cancer patients. Next, we used the online analysis website cBioPortal to analyze whether SNORA72 was related to certain stemness markers in ovarian cancer patients. The analysis results showed that the expressions of SNORA72 and c-Myc had co-expression characteristics (*P* < 0.001; Figure 5B). We further used the R2: Genomics Analysis and Visualization Platform to analyze the expression correlation between SNORA72 and c-Myc and also found a significantly positive relationship between SNORA72 and c-Myc expressions (*R* = 0.227, *P* = 1.12e^–04^; Figure 5C).

### SNORA72 Promotes Stemness of OCSCs by Regulating the Notch1/c-Myc Pathway

**FIGURE 6:**
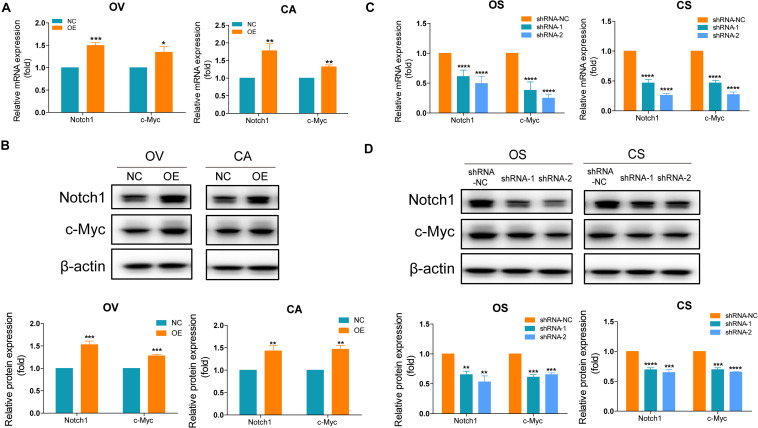
SNORA72 induces the stemness of ovarian cancer by activating the Notch1/c-Myc pathway. **(A)** Relative mRNA expressions of Notch1 and c-Myc to β-actin, as an endogenous control, analyzed by quantitative reverse transcription PCR (qRT-PCR) in OVCAR-3 (OV) and CAOV-3 (CA) cells after transfection of the transfected (OE) or control (NC) plasmids for 48 h. The expression level in OV and CA cells after transfection of NC plasmids was set as 1. **(B)** Relative protein expressions of Notch1 and c-Myc to β-actin analyzed by Western blot in OV and CA cells after transfection of the OE or NC plasmids for 48 h. The expression level in OV and CA cells after transfection of NC plasmids was set as 1. **(C)** Relative mRNA expressions of Notch1 and c-Myc to β-actin analyzed by qRT-PCR in OVCAR-3 spheroid (OS) and CAOV-3 spheroid (CS) cells after transfection of the shRNA-1, shRNA-2, or shRNA-NC plasmids for 48 h. The expression level in OS and CS cells after transfection of shRNA-NC plasmids were set as 1. **(D)** Relative protein expressions of Notch1 and c-Myc to β-actin analyzed by Western blot in OS and CS cells after transfection of the shRNA-1, shRNA-2, or shRNA-NC plasmids for 48 h. The expression level in OS and CS cells after transfection of shRNA-NC plasmids was set as 1. *P < 0.05, **P < 0.01, ***P < 0.001, and ****P < 0.0001.

**FIGURE 7:**
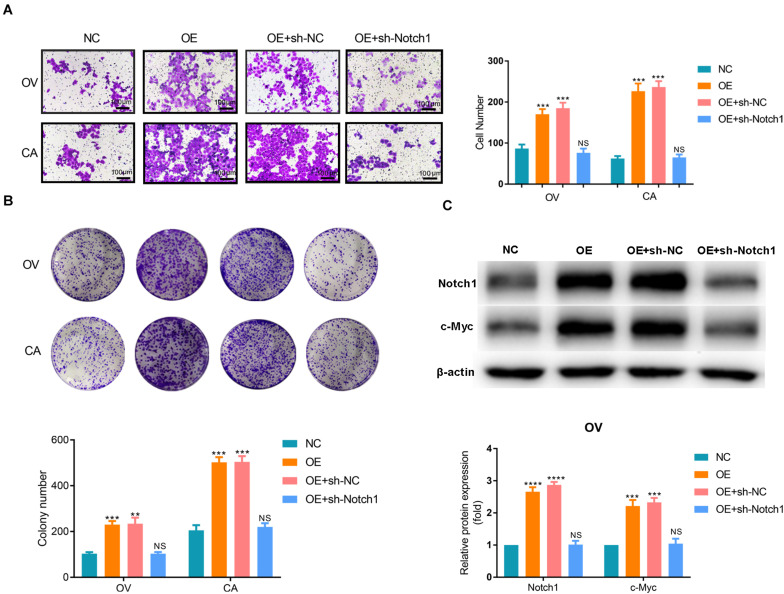
SNORA72-NC, SNORA72-OE, SNORA72-OE+sh-NC, or SNORA72-OE+sh-Notch1 plasmids were, respectively, transfected in OVCAR-3 (OV) or CAOV-3 (CA) cells. **(A)** Migration abilities of OV and CA cells by the Transwell assay. **(B)** The self-renewal abilities of OV and CA cells were measured by colony formation assays. **(C)** Relative protein expressions of Notch1 and c-Myc to β-actin analyzed by Western blot in OV cells. **P < 0.01, ***P < 0.001, and ****P < 0.0001.

Based on the results of the bioinformatics analyses, we assume that SNORA72 may regulate c-Myc expression to maintain the stemness of OCSCs. c-Myc is a major target gene of the Notch pathway. Therefore, we measured the expression changes of c-Myc and Notch1 after inferring SNORA72 expression. We found that the mRNA and protein levels of Notch1 and c-Myc expressions were both markedly elevated in OV and CA cells transfected with SNORA72-OE plasmids compared with the cells transfected with NC plasmids (Figures 6A,B). Conversely, the knockdown of SNORA72 in OS and CS cells notably decreased the mRNA and protein expressions of Notch1 and c-Myc (Figures 6C,D). Next, we respectively transfected SNORA72-NC, SNORA72-OE, SNORA72-OE+sh-NC, or SNORA72-OE+sh-Notch1 plasmids in OV and CA cells and detected the migration, self-renewal abilities, and protein expressions of Notch1 and c-Myc. We found that silencing Notch1 could inverse the effects of SNORA72 overexpression on the migration, self-renewal abilities, and protein expressions of Notch1 and c-Myc in OV and CA cells (Figures 7A–C). Thus, these findings suggest that SNORA72 can induce the stemness of ovarian cancer by activating the Notch1/c-Myc pathway.

## Discussion

Ovarian cancer is a malignant cancer with a high migration and invasive potential and a low survival rate since many patients are at an advanced stage when diagnosed (Vaughan et al., 2011). CSCs are considered the source of cancer cells that arise and are responsible for the metastasis and relapse of cancer (Peiris-Pages et al., 2016). Therefore, exploring the mechanisms of cancer development and searching for therapeutic strategies for the effective eradication of cancer by targeting CSCs have attracted much attention (Eun et al., 2017; Shibata and Hoque, 2019).

A growing body of evidence has shown that snoRNAs have oncogenic or tumor-suppressive functions in various cancers (Stepanov et al., 2015; Liang et al., 2019). It has been reported that SNORA42 could have an oncogenic role in lung tumorigenesis (Mei et al., 2012) and could enhance prostate cancer cell viability and migration (Yi et al., 2018). On the other hand, Zheng et al. demonstrated that SNORD78 was upregulated in cancer stem-like non-small-cell lung carcinoma (NSCLC) cells, which showed that snoRNAs were required for the self-renewal of CSCs (Zheng et al., 2015). In the present study, we found a higher expression of SNORA72 in OS cells with OCSC-like characteristics compared to the parental OV cells by non-coding RNA-ChIP analysis. Further qRT-PCR analysis also demonstrated the abnormally high expression of SNORA72 in OS and CS cells, suggesting that SNORA72 may promote the stemness of ovarian cancer cells.

Cancer stem cells have self-renewal abilities and increased migration abilities (Lytle et al., 2018). We found that the overexpression of SNORA72 promoted the self-renewal and migration abilities of OV and CA ovarian cancer cells by a plate clone formation assay, wound-healing assay, and Transwell assay. Conversely, silencing SNORA72 reduced the self-renewal and migration abilities of OS and CS cells. CD133 is one of the OCSC markers (Curley et al., 2009; Liou, 2019), and Nanog and Oct4 are also major CSC markers (Zhang et al., 2013; Iv Santaliz-Ruiz et al., 2014; He et al., 2019). In this study, SNORA72 overexpression elevated the expressions of CD133, Nanog, and Oct4 in OV and CA cells, while SNORA72 knockdown decreased the expressions of these stemness markers in OCSCs. Thus, SNORA72 plays an important role in the stemness transformation and maintenance of ovarian cancer cells.

NOTCH signaling is a major cell communication system during organ development and plays important roles in oncogenesis and stem regulation of cells (Andersson et al., 2011). The activation of NOTCH1, one of four main NOTCH receptors, drives the metastasis of ovarian carcinoma cells (Wieland et al., 2017) and the resistance of OCSCs (Islam and Aboussekhra, 2019). C-Myc, a CSC survival factor, controls the balance between the self-renewal and differentiation of stem cells (Wilson et al., 2004). C-Myc is also an important transcriptional target of Notch1 signaling in various cancers (Klinakis et al., 2006). As in our previous study, Notch1 and c-Myc were highly expressed in OS cells (Zhu et al., 2019). Interestingly, we identified a positive relationship between SNORA72 and c-Myc mRNA expressions in patients with ovarian cancer by R2: Genomics Analysis and Visualization Platform analysis. We further demonstrated that SNORA72 significantly increased the protein expressions of Notch1 and c-Myc in OV and CA cells. In contrast, knocking down SNORA72 notably reduced their expressions in OS and CS cells.

In summary, we showed that SNORA72 was markedly upregulated in OCSCs. SNORA72 promotes the self-renewal and migration of ovarian cancer cells and is vital for the maintenance of stemness in OCSCs. Additionally, we also demonstrated that SNORA72 induced the stemness transformation of ovarian cancer cells by activating the Notch1/c-Myc pathway. Our study provides crucial evidence that SNORA72 contributes to ovarian cancer development and will be helpful for developing a novel therapeutic strategy for improving ovarian cancer treatment efficiencies.

## Data Availability Statement

The datasets presented in this study can be found in online repositories. The names of the repository/repositories and accession number(s) can be found in the article/Supplementary Material.

## Author Contributions

MH, MW, LwZ, and RM contributed to the conception and design. LwZ, RM, MG, YZ, XL, WZ, LH, PS, and YF conducted the experiments and acquired data. LwZ, RM, MG, MH, and MW analyzed the data. LwZ, MH, MW, RM, YY, HM, and LnZ wrote, reviewed, and/or revised the manuscript. MH and MW supervised the study. All authors contributed to the article and approved the submitted version.

## Conflict of Interest

The authors declare that the research was conducted in the absence of any commercial or financial relationships that could be construed as a potential conflict of interest.

## Footnotes

1
http://kmplot.com/analysis/index.php?p=service&cancer=ovar

2
http://www.cbioportal.org/

3
https://hgserver1.amc.nl/cgi-bin/r2/main.cgi

## Abbreviations

CA: CAOV-3 cells
cDNA: complementary DNA
CRC: colorectal cancer
CS: CAOV-3 spheroid cells
CSCs: cancer stem cells
ncRNA: non-coding RNA
OCSCs: ovarian cancer stem cells
OV: OVCAR-3 cells
OS: OVCAR-3 spheroid cells
PDAC: pancreatic ductal adenocarcinoma
PFS: progression-free survival
qRT-PCR: quantitative real-time PCR
shRNA: short hairpin RNA
snoRNAs: small nucleolar RNAs
SNORDs: C/D box snoRNAs
SNORAs: H/ACA box snoRNAs.
