# Corrigendum: SNORA72 activates the Notch1/c-Myc pathway to promote stemness transformation of ovarian cancer cells

**DOI:** 10.3389/fcell.2022.819798

**Published:** 2022-08-26

**Authors:** Liwen Zhang, Rong Ma, Mengcong Gao, Yanyun Zhao, Xuemei Lv, Wenjing Zhu, Li Han, Panpan Su, Yue Fan, Yuanyuan Yan, Lin Zhao, Heyao Ma, Minjie Wei, Miao He

**Affiliations:** ^1^ Department of Pharmacology, School of Pharmacy, China Medical University, Shenyang, China; ^2^ Liaoning Key Laboratory of Molecular Targeted Anti-Tumor Drug Development and Evaluation, China Medical University, Shenyang, China; ^3^ Liaoning Cancer Immune Peptide Drug Engineering Technology Research Center, China Medical University, Shenyang, China; ^4^ Key Laboratory of Precision Diagnosis and Treatment of Gastrointestinal Tumors, Ministry of Education, China Medical University, Shenyang, China

**Keywords:** SNORA72, ovarian cancer stem cells (OCSCs), Notch1, c-Myc, stemness

In the original article, there was a mistake in Figure 1 as published. The transwell picture for CA in Figure 1C was mistaken and misused. The corrected Figure 1 appears below.

**FIGURE 1:**
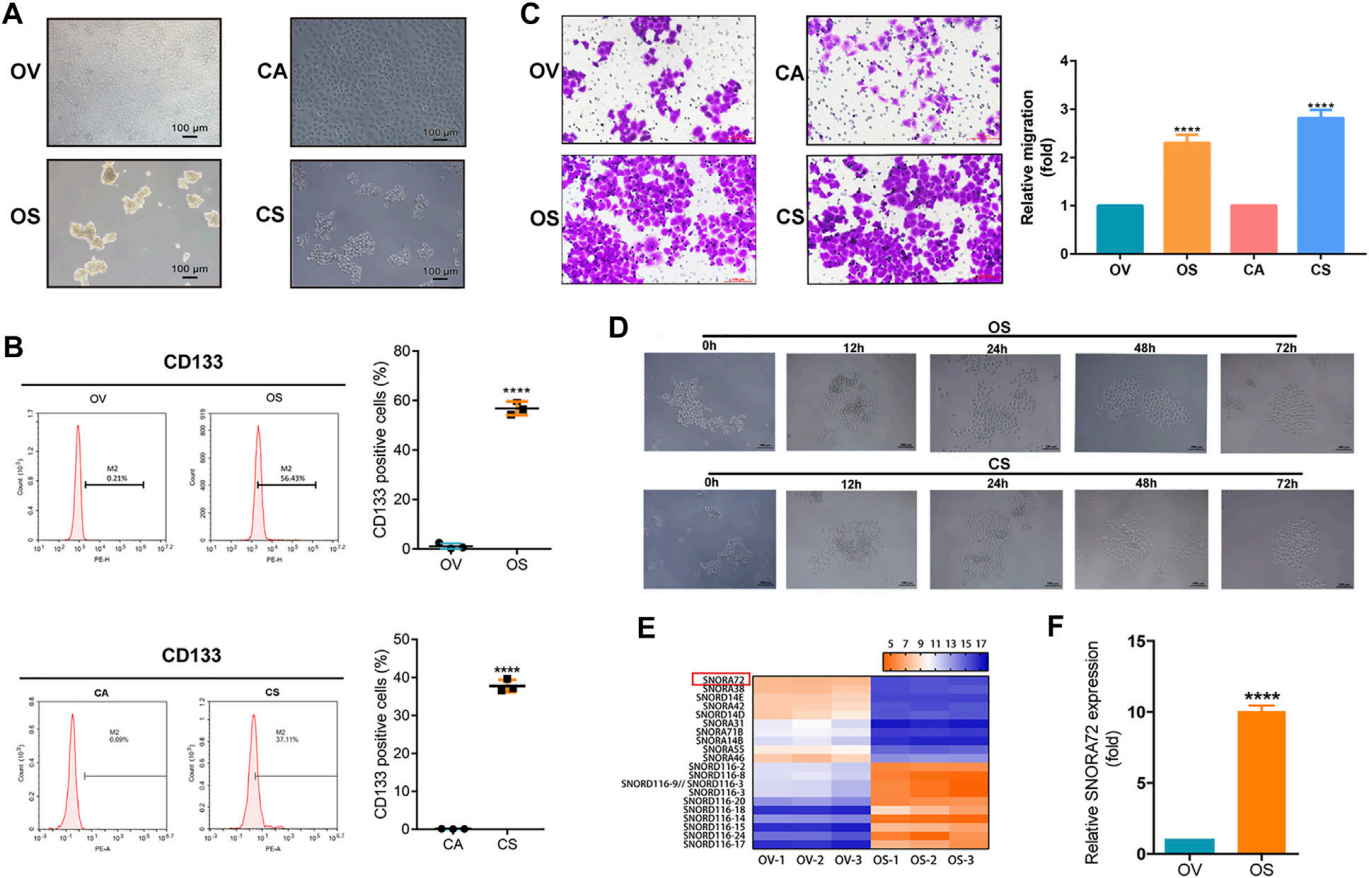
SNORA72 is overexpressed in ovarian cancer stems cells (OCSCs). **(A)** Morphology of OVCAR-3 (OV), OVCAR-3 spheroid (OS), CAOV-3 (CA), and CAOV-3 spheroid (CS) cells shown under a microscope (×10). **(B)** Expression of CD133 detected by flow cytometry in OV vs. OS and in CA vs. CS cells. **(C)** Migration abilities of OV, OS, CA, and CS cells by Transwell assay. **(D)** Differentiation morphology of OS and CS cells at 0, 12, 24, 48, and 72 h. **(E)** Hierarchical clustering analysis of small nucleolar RNA (snoRNA) expression from non-coding RNA-ChIP data in OV and OS cells. Red, higher expression levels; green, lower expression levels. **(F)** Relative SNORA72 expression to U6, as an endogenous control, analyzed by qRT-PCR in OV and OS cells. The SNORA72 expression in OV cells was set as 1. Data are shown as the mean ± SD from three independent experiments. **p < 0.01, ****p < 0.0001.

The authors apologize for this error and state that this does not change the scientific conclusions of the article in any way. The original article has been updated.

