# Supplementary material for: SNORA72 Activates the Notch1/c-Myc Pathway to Promote Stemness Transformation of Ovarian Cancer Cells
Source: Front Cell Dev Biol. 2020 Nov 3;8:583087. doi: 10.3389/fcell.2020.583087 (PMC7669759; doi:10.3389/fcell.2020.583087)
Supplement: Supplementary file 2 [file Table_1.DOCX]

Table1. Limited gradient dilution analysis experiment for OV, OS, CA and CS

|  | OV | | | | OS | | | | CA | | | | CS | | | |
| --- | --- | --- | --- | --- | --- | --- | --- | --- | --- | --- | --- | --- | --- | --- | --- | --- |
| Dilution ratio | 1/1 | 1/2 | 1/4 | 1/8 | 1/1 | 1/2 | 1/4 | 1/8 | 1/1 | 1/2 | 1/4 | 1/8 | 1/1 | 1/2 | 1/4 | 1/8 |
| Cells/well | 200 | 100 | 50 | 25 | 200 | 100 | 50 | 25 | 200 | 100 | 50 | 25 | 200 | 100 | 50 | 25 |
| Wells with colonies | 12 | 8 | 7 | 3 | 20 | 20 | 18 | 16 | 14 | 9 | 7 | 4 | 20 | 20 | 18 | 15 |
| Total colonies | 163 | 49 | 21 | 9 | 1215 | 559 | 235 | 107 | 176 | 54 | 25 | 11 | 1179 | 535 | 233 | 113 |
| Total cells | 4000 | 2000 | 1000 | 500 | 4000 | 2000 | 1000 | 500 | 4000 | 2000 | 1000 | 500 | 4000 | 2000 | 1000 | 500 |
| Colonies/total cells (%) | 4.07 | 2.45 | 2.1 | 1.8 | 30.37 | 27.95 | 23.5 | 21.4 | 4.4 | 2.7 | 2.5 | 2.2 | 29.48 | 26.75 | 23.3 | 22.6 |

Abbreviations: OV: OVCAR-3 cells; OS: OVCAR-3 spheroids cells; CA: CAOV-3 cells; CS: CAOV-3 spheroids cells
