# Supplementary figures and images for: SNORA72 Activates the Notch1/c-Myc Pathway to Promote Stemness Transformation of Ovarian Cancer Cells
Source: Front Cell Dev Biol. 2020 Nov 3;8:583087. doi: 10.3389/fcell.2020.583087 (PMC7669759; doi:10.3389/fcell.2020.583087)

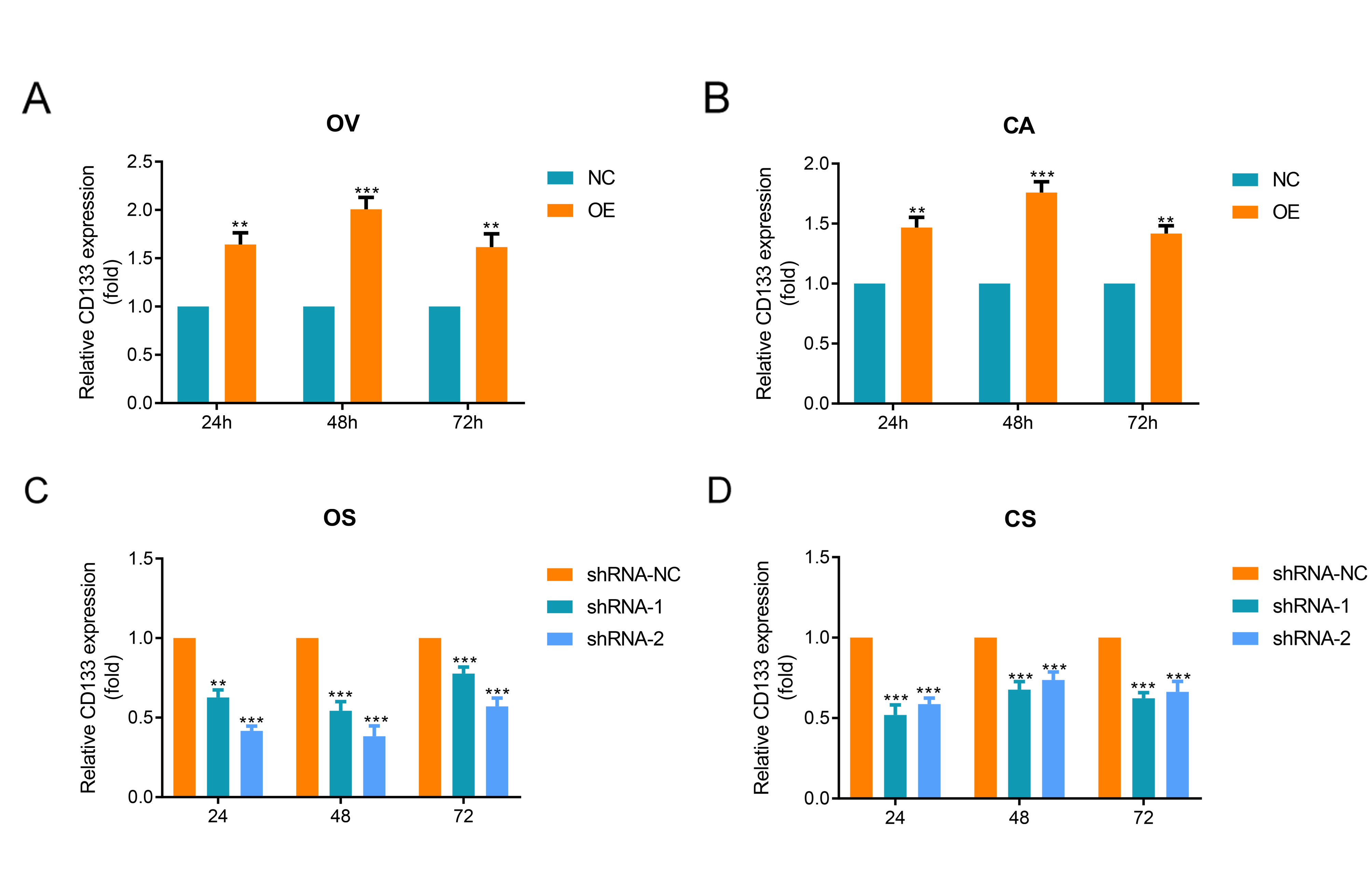

Supplement: Supplementary file 3 [file Image_1.tif]
